# Measuring emergent mechanical changes in cytoskeletal ensembles *in vitro* using QCM-D

**DOI:** 10.3389/fcell.2025.1616969

**Published:** 2025-08-07

**Authors:** Victoria N. Amari, Emily M. Kerivan, Dana N. Reinemann

**Affiliations:** ^1^Department of Biomedical Engineering, University of Mississippi, University, MS, United States; ^2^Department of Chemical Engineering, University of Mississippi, University, MS, United States

**Keywords:** actin, myosin, QCM-D, cytoskeleton, biomechanics, *in vitro* reconstitution

## Abstract

This work presents a protocol for using a quartz crystal microbalance with dissipation monitoring (QCM-D) to measure emergent cytoskeletal mechanics in reconstituted actomyosin systems. Cytoskeletal ensembles exhibit emergent behaviors that cannot be solely inferred from the properties of their constituent molecules. The underlying design principles and mechanisms governing this collective behavior remain poorly understood. Previous work suggests that cytoskeletal filaments, particularly actin, function as force sensors that regulate motor protein activity, influencing contraction, force generation, and cellular shape during movement. To investigate these emergent mechanics, we developed a novel approach utilizing QCM-D to study reconstituted actomyosin bundle systems. Our results demonstrate that QCM-D is capable of detecting viscoelastic changes in actomyosin bundles in response to molecular-scale perturbations, including variations in concentration, nucleotide state, and actin-binding affinity. These findings support the role of actin as a mechanical force-feedback sensor and establish QCM-D as a powerful, complementary technique to optical trapping and fluorescence imaging for probing cytoskeletal ensemble mechanics and motor protein interactions.

## Introduction

The cytoskeleton provides structure to the cell and facilitates important cellular tasks such as motility, intracellular transport, and cell division (Kerivan et al., 2024; Dumont and Prakash, 2014; Howard, 2001; Stewart et al., 2021). An interconnected network of actin filaments, microtubules, intermediate filaments, and all associated proteins make up the cytoskeleton (Kerivan et al., 2023; Al Azzam O. et al., 2022; Finer et al., 1994). When these pieces assemble, they determine the functional behavior and mechanical properties of a cell (Kerivan et al., 2024; Kerivan et al., 2023; Kaya et al., 2017; Wagoner and Dill, 2021). Cytoskeletal protein ensembles exhibit mechanics that cannot necessarily be determined from the sum of its constituents’ properties, and this emergent behavior is not well understood (Biolin, 2021; Walcott et al., 2012; Hilbert et al., 2013; Jegou and Romet-Lemonne, 2021). Understanding the design principles that drive such emergent behavior would aid in engineering blueprints of cells to better understand and predict cellular phenotypes under various conditions (Gagliardi et al., 2023; Liu et al., 2018; Koenderink et al., 2009; Lee et al., 2021). Interestingly, evidence has shown that cytoskeletal filaments may also act like force sensors and thus influence the behavior of motor proteins (Kushiro et al., 2016; Sun and Alushin, 2022; Greenberg and Moore, 2010; Liu et al., 2018). Myosin II’s specific ability to contract, generate force, and maintain cellular shape during movement depends on feedback mechanism provided by the actin network (Al Azzam et al., 2021; Stachowiak et al., 2012; Pollard et al., 1992). This force-feedback relationship is critical for cellular processes such as migration during development and tissue repair, while disruptions to these dynamics are often associated with pathological states (Tagaya, 2015; Greenberg and Moore, 2010; Debold et al., 2013).

Actin filaments and myosin II motors are key cytoskeletal proteins that work in conjunction to regulate the mechanical properties of cells (Luo et al., 2013; Dwyer et al., 2022; Isambert et al., 1995; Janmey et al., 1990). Actin filaments form dynamic, semi-flexible polymers that provide structural support and define cell shape (Luo et al., 2013; Dwyer et al., 2022; Isambert et al., 1995; Janmey et al., 1990). Myosin II, a motor protein, binds to and exerts force on actin filaments through the conversion of chemical energy into mechanical work via ATP hydrolysis (Al Azzam et al., 2021; Howard, 2001; Finer et al., 1994). While the molecular properties of actin and myosin are well-characterized, it is their collective behavior in ensembles that contributes to larger-scale cellular behavior (Gagliardi et al., 2023; Bianco et al., 2018; Al Azzam O. Y. et al., 2022; Ruegg et al., 2002; Jegou and Romet-Lemonne, 2021). Recent studies by Al Azzam et al. have shown that these ensembles exhibit force-dependent behavior controlled by intracellular mechanosensing and feedback regulation (Al Azzam et al., 2021; Al Azzam O. et al., 2022; Al Azzam O. Y. et al., 2022). Specifically, myosin II modulates its motility and force output based on local mechanical resistance, adapting to its environment to maintain appropriate cytoskeletal tension (Al Azzam O. Y. et al., 2022; Wagoner and Dill, 2021; Tyska et al., 1999). Using optical tweezers to probe reconstituted actomyosin bundles, these studies demonstrated that myosin ensembles may alter their activity depending on the stiffness and architecture of the surrounding actin network (Al Azzam et al., 2021; Al Azzam O. et al., 2022; Kaya and Higuchi, 2010; Molloy et al., 1995).

Among the methods traditionally available to study cytoskeletal mechanics, Quartz Crystal Microbalance with Dissipation Monitoring (QCM-D) has emerged as a powerful technique (Kerivan et al., 2023; Biolin Scientific, 2024; Biolin Scientific, 2017). Traditionally utilized for characterizing thin films and polymers, QCM-D measures changes in resonance frequency (∆f) and energy dissipation (∆D) to provide insights into mass accumulation and viscoelastic changes on a piezoelectric sensor surface (Kerivan et al., 2024; Gagliardi et al., 2023; Dixon, 2008). For an extensive review of QCM-D function, previous cytoskeletal studies, and data interpretation, see Kerivan et al. (Kerivan et al., 2023) Briefly, changes in frequency (∆f) reflect mass loading, where a decrease indicates accumulation and an increase indicates removal, while changes in dissipation (∆D) reflect viscoelastic alterations, with increased dissipation signifying reduced film rigidity (Biolin, 2021; Biolin, 2021). Additional studies have suggested that QCM-D can be used to characterize protein conformational changes and responses to ligand binding (Kwon and Dodge, 2015; Albet-Torres et al., 2007; Albet-Torres et al., 2010). This dual measurement capability makes QCM-D uniquely suited for real-time studies of biomolecular assemblies, offering a quantitative lens into dynamic processes such as protein binding, structural rearrangements, and mechanical alterations (Kerivan et al., 2024; Kerivan et al., 2023; Albet-Torres et al., 2010; Hanson et al., 2005). A growing area of research has explored the use of QCM-D to study cell adhesion, detachment, and biomolecular interactions at nano-bio interfaces, further supporting its application in cytoskeletal mechanics (Biolin, 2021; Tagaya, 2015; Biolin, 2021; Suda et al., 2004). For example, QCM-D has been used to track protein-material interactions and changes in cellular behaviors in order to present how different cytoskeletal components respond to environmental change (Tonda-Turo et al., 2018; Kushiro et al., 2016; Hanson et al., 2005). Additionally, changes in ionic strength have been shown to dramatically alter actomyosin network stiffness through salt-mediated stiffening and resculpting mechanisms (Biolin, 2021; Bianco et al., 2018; Gurmessa et al., 2021; Suda et al., 2004).

To uncover the mechanisms that drive intracellular cytoskeletal function, an innovative approach was developed, leveraging QCM-D to measure mechanical changes within reconstituted actomyosin bundles (Kerivan et al., 2024; Kerivan et al., 2023). The QCM-D approach focuses on detecting and quantifying changes in the viscoelastic properties of actomyosin bundles in response to molecular-level changes (Kerivan et al., 2024; Kwon and Dodge, 2015). Previous work by Kerivan and Amari et al. demonstrates that QCM-D can sensitively detect variations in actomyosin viscoelasticity induced by changes in nucleotide state (ATP vs. ADP) and the presence of actin-binding or crosslinking proteins (Kerivan et al., 2024; Biolin, 2021; Ghodsi and Kazemi, 2012). These results further support the role of actin filaments as mechanical force-feedback sensors (Kushiro et al., 2016; Tagaya, 2015; Pollard et al., 1992). The stiffness and structure of actin filaments depend on their nucleotide state and the behavior of its constituent binding proteins (Bianco et al., 2018; Debold et al., 2013; Bidone et al., 2015; Ghodsi and Kazemi, 2012; Ozeki et al., 2009). Myosin II motor activity introduces contractile forces that significantly alter the viscoelastic response of actin filaments, not only through force production but also via transient binding and unbinding cycles (Al Azzam et al., 2021; Kushiro et al., 2016; Hilbert et al., 2013). Myosin II exhibits a strongly-bound state with actin in the presence of ADP and a weakly-bound state when bound to ATP (Stachowiak et al., 2012; Debold et al., 2013). The number of engaged myosin heads, governing the number of actin-myosin cross bridges, regulates bundle stiffness in real time (Stachowiak et al., 2012; Debold et al., 2013). As more myosin heads bind, they generate increased crosslinking and tension resulting in a stiffer, more mechanically resistant actin network (Al Azzam et al., 2021; Kushiro et al., 2016; Hilbert et al., 2013). Conversely, unbinding events and reduced motor activity lead to relaxation and softening of the filament assembly (Al Azzam et al., 2021; Kushiro et al., 2016; Hilbert et al., 2013). These changes are reflective of the dynamic mechanical activity inherent to actomyosin networks (Kerivan et al., 2023; Kushiro et al., 2016; Bianco et al., 2018). The ability of QCM-D to quantitatively measure these mechanical alterations provides a platform for identifying the molecular mechanisms underlying force transmission within the actomyosin cytoskeleton (Kerivan et al., 2023; Bianco et al., 2018; Walcott et al., 2012; Hilbert et al., 2013; Wagoner and Dill, 2021).

Compared to traditional techniques for studying cytoskeletal mechanics, QCM-D offers distinct advantages (Kerivan et al., 2024). Unlike microscopy-based methods, QCM-D does not require fluorescent labeling, enabling real-time measurements without the risk of photobleaching (Kerivan et al., 2024; Kerivan et al., 2023; Gagliardi et al., 2023; Biolin Scientific, 2024; Biolin Scientific, 2017). Furthermore, the nanoscale sensitivity of QCM-D allows for the detection of subtle mechanical changes that may elude bulk rheological methods or single-molecule force spectroscopy (Kerivan et al., 2023; Gagliardi et al., 2023; Dwyer et al., 2022). This makes QCM-D particularly well-suited for exploring the emergent properties of cytoskeletal ensembles, where molecular interactions and coordination give rise to collective behaviors that are difficult to capture with other techniques (Kerivan et al., 2023; Kushiro et al., 2016; Stachowiak et al., 2012; Ghodsi and Kazemi, 2012). In actomyosin systems, QCM-D enables simultaneous measurements of mass accumulation (via frequency shifts) and changes in viscoelastic properties (via dissipation shifts). These parameters provide real-time insight into dynamic events such as myosin binding and unbinding, changes in cross-bridge density, and structural transitions within the actin network (Kerivan et al., 2024; Albet-Torres et al., 2010; Bidone et al., 2015). This level of detail allows researchers to distinguish between passive filament adsorption, active motor engagement, and mechanically regulated bundle formation (Kerivan et al., 2023; Kushiro et al., 2016; Stachowiak et al., 2012; Ghodsi and Kazemi, 2012). For biophysicists, these measurements help bridge the gap between molecular-scale interactions and macroscale mechanical outputs, advancing our understanding of how cytoskeletal systems integrate force generation, stiffness modulation, and biochemical signaling in real time (Kerivan et al., 2023; Gagliardi et al., 2023; Stachowiak et al., 2012; Debold et al., 2013; Luo et al., 2013; Pollard et al., 1992).

The protocol developed for using QCM-D to study actomyosin bundles includes detailed steps for sensor cleaning, solution preparation, and experimental procedures for measuring frequency and dissipation changes (Kerivan et al., 2024; Kerivan et al., 2023; Dixon, 2008). These steps are designed to yield reproducible data on the viscoelastic properties of actomyosin networks, emphasizing how molecular-level changes influence their mechanical behavior (Kerivan et al., 2024; Kerivan et al., 2023; Gagliardi et al., 2023; Dixon, 2008). Importantly, the assay is highly customizable. Investigators can tune filament composition, motor isoforms, crosslinking protein, and buffer conditions to construct cytoskeletal assemblies that reflect the mechanical complexity of an *in vivo* system (Kerivan et al., 2023; Dwyer et al., 2022; Gurmessa, 2023). This flexibility and customizability not only provide insights into actomyosin bundle mechanics but also serves as a blueprint for investigating other biomolecular systems where mechanical properties play a critical role (Kerivan et al., 2023; Dwyer et al., 2022; Gurmessa, 2023). In addition to actomyosin, the QCM-D has been used to probe mechanics of diverse cytoskeletal systems such as microtubule-kinesin networks, intermediate filaments, or composite systems where multiple filament types are integrated (Luo et al., 2013; Gurmessa, 2023; Kucera et al., 2021; Lee et al., 2021). For example, investigators may use this approach to study mechanical coupling between actin and microtubule during cell migration (Luo et al., 2013; Gurmessa, 2023; Kucera et al., 2021; Lee et al., 2021). By elucidating the factors that regulate reconstituted protein bundle mechanics, this methodology can aid investigators in understanding the design principles underlying cytoskeletal organization and function (Koenderink et al., 2009; Liu et al., 2018; Pollard et al., 1992).

## Materials and equipment

**Table:** 

Reagent	Source	Identifier
Chemicals		
Tris-HCl	Promega	H5121
CaCl_2_	Fisher Scientific	10035-04-8
KCl	Fisher Scientific	7447-40-7
MgCl_2_	Fisher Scientific	7791-18-6
Adenosine 5′Triphosphate Disodium Salt Trihydrate	Fisher Scientific	987-65-5
Adenosine 5′-Diphosphate Sodium Salt	Sigma Aldrich	20398-34-9
DL-1,4-dithiothreitol	Thermo Scientific	578517
KOH	Sigma Aldrich	1310583
NH_4_OH	Thermo Scientific	255210010
H_2_O_2_	Fisher Scientific	7722841/7732185
Poly-L-lysine (PLL)	Sigma Aldrich	25988630
Blotting Grade Blocker (Casein)	Bio-Rad Laboratories	1706404
Cytoskeleton		
Skeletal Myosin II	Cytoskeleton	MY02
Skeletal Actin	Cytoskeleton	AKL99-C
Rhodamine Phalloidin	Cytoskeleton	PHDR1
Fluorescence Microscope		
Optical Trap*	Bruker/JPK	NanoTracker2
QCM-D		
Qsense Analyzer	Biolin Scientific	
QSense Qsoft Software	Biolin Scientific	
Qsense Dfind Software	Biolin Scientific	
Peristaltic Pump	Biolin Scientific	Ismatec IPC-N 4
QCM-D Flow Module	Biolin Scientific	QFM401
Regular Tygon Tubing	Biolin Scientific	QCS007
Gold QSensors 5 Pack	Biolin Scientific	QSX301
QSense Liquid Handling Set	Biolin Scientific	QLH401
Tube Cutting Device from Installation Kit	Biolin Scientific	
Angled Tweezers		
Other		
Analog Vortex	Fisher Scientific	2215414
mySPIN 6 Mini Centrifuge	Thermo Scientific	75004061
SureOne Micropoint Pipette Tips	Fisher Scientific	2707407
SureStack Pipette Tips	Fisher Scientific	2100503
SureOne Micropoint Pipette Tips	Fisher Scientific	2707441
Non-Latex Gloves	Fisher Scientific	191301597B
Parafilm PM-992 Roll	Fisher Scientific	501367664
Kimberly-Clark Professional Kimwipes	Fisher Scientific	S47299
Analytical Balance ME104E/A04	Mettler Toledo	30046415
Cimarec Basic Stirring Hotplates	Thermo Scientific	SP194715
Plastic pH Indicator Strips (5.0-9.0)	Fisher Scientific	13640519

*Any fluorescence microscope should work.

## Procedure

### Reconstitution of cytoskeletal proteins

Before performing the QCM-D experiments, the necessary cytoskeletal components must be prepared. This protocol outlines the steps for reconstituting skeletal myosin II and polymerizing actin filaments. Proper preparation of these proteins ensures reproducibility in the subsequent QCM-D measurements.

### Buffers

#### Concentrated KOH

1. In a 50 mL Falcon tube add approximately 5 KOH pellets
2. Fill the Falcon tube to 50 mL with DI water
3. Mix well

#### Solution T

1. In a 50 mL Falcon tube add 3.94 g Tris-HCl and 0.147 g CaCl_2_
  a. 0.5 M Tris-HCl and 0.02 M CaCl_2_
2. Add DI H_2_O to make a total volume of 50 mL and mix well
3. Label Solution T and store at 4°C

#### TC

1. Combine in a 100 mL jar
  a. 80 mL DI water
  b. 3 mL Solution T
    i. 0.5 M Tris-HCl and 0.02 M CaCl_2_
2. Mix well
3. Adjust pH to eight by adding small volumes of the concentrated KOH
4. Add DI water to make the final volume equal 100 mL and verify the pH
  a. Adjust pH as necessary with the concentrated KOH
  b. Will add an extra 17 mL between the KOH adjustment and the filling to 100 mL
5. Label as TC and store at 4°C

#### FC

1. Combine in a 100 mL jar
  a. 85 mL DI water
  b. 10 mL Solution T
    i. 0.5 M Tris-HCl and 0.02 M CaCl_2_
  c. 3.7249 g KCl
    i. 0.526 M
  d. 0.0403 MgCl_2_
    i. 0.02 M
2. Mix well
3. Adjust to 7.5 pH using small amounts of concentrated KOH
4. Add DI H_2_O to make the total volume 100 mL and verify the pH
5. Label as FC and store at 4°C

#### 100 mM ATP

1. Combine in a 15 mL centrifuge tube
  a. 0.061 g solid ATP from the −80°C freezer
    i. Solid ATP M.W. is 605.2 g/mol
  b. 1000 μL FC
2. Mix well
3. Adjust the pH to seven using small amounts of concentrated KOH
4. Make 1 mL aliquots
5. Label ATP and store at −80°C
  a. Concentration is 0.101 M

#### 10 mM ATP

1. Combine 1 mL of the 100 mM ATP +9 mL FC buffer
  a. This is a 10x dilution
  b. Provides 10 mL of 10 mM ATP

#### 100 mM ADP

1. Combine in a 15 mL centrifuge tube
  a. 0.0471 g solid ADP from the −80°C freezer
    i. Solid ADP M.W. is 471.17 g/mol
  b. 1000 μL FC
2. Mix well
3. Adjust the pH to seven using small amounts of concentrated KOH
4. Make 1 mL aliquots
5. Label ATP and store at −80°C
  a. Concentration is 0.101 M

#### 10 mM ADP

1. Combine 1 mL of the 100 mM ADP +9 mL FC buffer
  a. This is a 10x dilution
  b. Provides 10 mL of 10 mM ADP

#### 1 M DTT

1. Combine in a 15 mL centrifuge tube
  a. 0.308 g DTT
    i. M.W. of DTT is 154.24 g/mol
  b. 2 mL DI H_2_O
2. Mix well and dissolve completely
3. Label 1M DTT and store at −80°C
  a. Concentration is 0.998 M

#### 100 mM DTT

1. Combine 1 mL 1 M DTT +9 mL DI water
  a. This provides 10 mL of the 100 mM DTT
2. Aliquot into 5 mL, label 100 mM DTT and store at −80°C

#### 50 mM DTT

1. Combine 5 mL 100 mM DTT +5 mL DI water
  a. This provides 10 mL of the 50 mM DTT
2. Aliquot into 1 mL, label 50 mM DTT and store at −80°C

#### General actin buffer (GAB)

1. Combine in a 100 mL jar
  a. 97 mL of TC
  b. 2 mL of 10 mM ATP
  c. 1 mL of 50 mM DTT
2. Mix Well
3. Label GAB and store at 4°C

#### Actin polymerization buffer (APB)

1. Combine in a 100 mL jar
  a. 91 mL of FC
  b. 5 mL of 10 mM ATP
  c. 4 mL of 50 mM DTT
2. Mix well
3. Label APB and store at 4°C

#### ADP wash

1. Combine in a 15 mL falcon tube
  a. 14 mL and 550 uL TC
  b. 300 μL 10 mM ADP
  c. 150 μL 50 mM DTT

#### PLL in GAB

1. Combine 30 μL of Poly-l-lysine (PLL) and 3 mL GAB in 15 mL falcon tube
2. Label PLL in GAB and store at 4°C

#### Casein in GAB

1. Combine 0.1 mg of casein and 10 mL GAB in 15 mL falcon tube
2. Label Casein in GAB and store at 4°C

### Reconstitution of proteins

#### Skeletal myosin II

1. Centrifuge the myosin tube briefly
2. Make 1 mM DTT in RO water
3. Add 100 μL of 1 mM DTT to the myosin
4. Leave the tube at room temperature for 2 h while occasionally gently tapping the tube to disperse the myosin powder
5. Gently pipette the myosin 3-4 times with a pipette set at 100 μL
  a. Avoid bubbles or mixing too harshly because the bubbles will oxidate the myosin, inactivating the protein
6. Aliquot the myosin into 3 μL
7. Label Myosin II
8. In dry ice, snap freeze the aliquots and store at −80°C
  a. This is now 10 mg/mL myosin II
  b. Concentration of 20.75 μM

#### Skeletal actin

1. Combine 100 μL DI H_2_O and 1 mg of rabbit skeletal actin
2. Mix well by gently mixing with pipette tip
3. Store at −80°C

### Actin polymerization and rhodamine labelling

Note: Do this protocol the day before actin imaging and QCM-D experiments.

1. Get 5 μL of stock skeletal actin
2. Add 50 μL of GAB
3. Mix gently by pipetting up and down
4. Incubate on ice for 1 h
5. Polymerize actin into filaments by adding 5.5 μL APB to solution
6. Mix gently by pipetting up and down
7. Incubate on ice for 20 min
  a. The actin is now polymerized
8. Add 5 μL rhodamine-labeled phalloidin
9. Mix gently by pipetting up and down
10. Incubate on ice for 1 h in the ice in the dark
  a. The actin is now stabilized
11. Label rhod-actin filament, wrap in aluminum foil and store in the dark at 4°C.

Note: Agitation must be minimized during handling to preserve actin filament integrity.

### Verifying actin polymerization using fluorescence microscopy

#### Flow cell for imaging the sample

1. Obtain a microscope slide and an etched coverslip
2. Prepare a PLL solution by adding 30 mL of 100% ethanol and 200 μL PLL to a 50 mL falcon tube
3. Place the coverslip into the PLL solution and allow to incubate in the solution for 15 min
4. Remove the coverslip with tweezer, taking care to only touch the edges, and then grasp it with a gloved hand on the edges
5. Dry the coverslip with filtered air until all the solution has evaporated from the surface
6. Place the coverslip into a humidity chamber while preparing the microscope slide
  a. A humidity chamber can be made by adding water to an empty pipette tip box and covering the lid with aluminum foil to block light
7. Place double sided tape in the center of plate to make a channel about 3 mm apart
8. Tear off the excess tape that hangs off the edges
9. Take the coverslip from the humidity chamber and add perpendicular on the double sided tape
  a. This should create a T like shape
10. Use a small tube to press air out from under the tape by pressing the tube on coverslip and carefully rolling out the air
  a. The tape should be transparent with no bubbles present when all the air is out

#### Confirm actin polymerization

1. Add 15 μL of 200x diluted rhodamine labeled actin to the flow cell channel
  a. Draw the actin through with a kim wipe at the other end of the channel but do not allow the channel to run dry
2. Incubate in a humidity chamber inside a 4°C fridge for 20 min
3. Wash the channel with 30 μL APB
4. Incubate in a humidity chamber inside a 4°C fridge for 10 min
5. Seal the open ends of the flow cell with nail polish to prevent evaporation
6. Confirm actin filament morphology using any fluorescence microscope with the appropriate fluorophores and laser lines or filters for excitation

### Preparing cytoskeletal reagents for QCM-D

Once actin filament polymerization is confirmed using fluorescence microscopy, the reagents are ready to be used in QCM-D measurements. Actin and myosin should only be diluted to their final working concentrations on the day of the experiment to preserve protein functionality. The exact concentrations may vary depending on the specific questions being investigated, but the conditions described below reflect those used to obtain the results presented in the results section.

Note: Polymerized actin should be used within 7 days of preparation and stored at 4°C in buffer to preserve filament integrity. Avoid repeatedly freezing and thawing and minimize mechanical agitation to prevent filament shearing.

Note: Myosin should be stored at −80°C and thawed on ice before use. Avoid refreezing thawed aliquots.

#### Actin dilution in GAB

1. Combine 6 μL rhodamine-labelled actin filaments and 3 mL GAB in a 15 mL falcon tube
2. Label Actin in GAB and store in ice to go to QCM
  a. Do this procedure twice for the Actomyosin Assay in the QCM-D

#### Myosin dilution in GAB

1. Combine in a 15 mL falcon tube
  a. 30 μL of Myosin II
  b. 3 mL of GAB
2. Label Myosin in GAB and store at in ice to go to QCM

Note: Store the diluted solutions on ice and go perform the QCM-D experiments immediately.

### QCM-D sensor and equipment preparation

**FIGURE 1:**
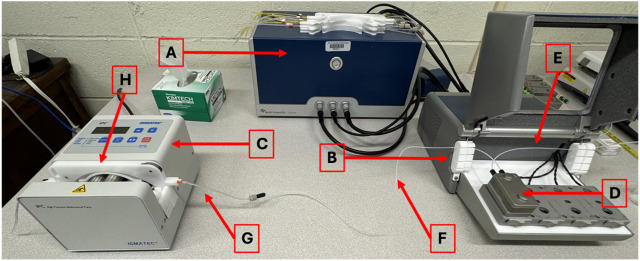
Biolin Scientific QCM-D Set-Up. **(A)** QCM-D power supply **(B)** QCM-D **(C)** Peristaltic pump **(D)** Flow module **(E)** Inlet tubing **(F)** Outlet tubing **(G)** Tygon tubing for pump **(H)** Cassette for pump tubing

Before running a QCM-D experiment (equipment shown in Figure 1), it is essential to prepare the reagents and sensor surfaces in a consistent, reproducible manner. Typically, actin is polymerized, and myosin is reconstituted the day before the experiment. On the morning of the experiment, both proteins are diluted to their final concentrations, and the QCM-D sensors are cleaned to ensure a pristine surface for reliable adsorption and interaction measurements. The cleaning process is critical as any residual contamination can affect frequency and dissipation readings.

#### Sensor cleaning

The first step of the experiment day is to clean the sensor with a base piranha solution. Base piranha solution is a 5:1:1 mixture of RO water, 25% ammonia (NH_3_), 30% hydrogen peroxide (H_2_O_2_).

Caution: Piranha solution should be handled with appropriate personal protective equipment (PPE) in a fume hood.

The protocol is as follows.

**FIGURE 2:**
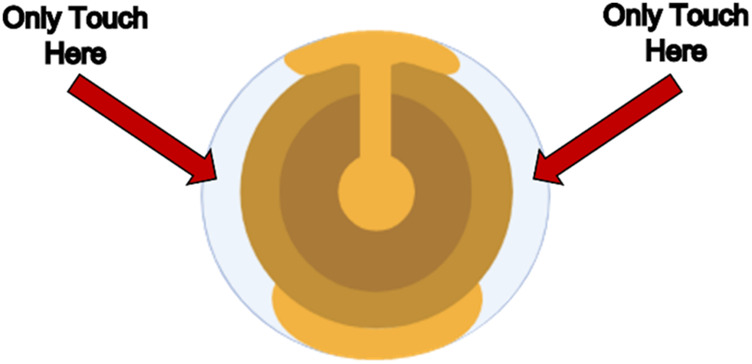
Biolin Scientific Gold-Coated Quartz Sensor. The sensor should only be touched on the indicated quartz crystal edges to avoid scratching the gold coating, which would interfere with frequency and dissipation measurements.

1. Heat 20 mL of RO H_2_O to 80°C in a 100 mL beaker
2. After 80°C is reached, add
  a. 4 mL NH_3_ + 4 mL H_2_O_2_
3. While allowing temperature to adjust back to 80°C, carefully place sensors in durable dipping/cleaning stand
  a. As seen in Figure 2, be careful to only touch the quartz portion of the sensor so as to not scratch the gold coating.
4. Place stand in the beaker
5. Allow sensors to stay in piranha solution for 5–10 min (or until bubbles appear on the sensors)
6. Place sensor holder on a paper towel beside the sink
7. Rinse each sensor individually with RO water
  a. Keep sensor over the paper towel in case it falls
8. Immediately dry with nitrogen or air
  a. Keep sensor over the paper towel in case it falls
9. Immediately store the cleaned sensor in a sensor case to avoid dust and contaminant exposure

#### QCM module preparation

**FIGURE 3:**
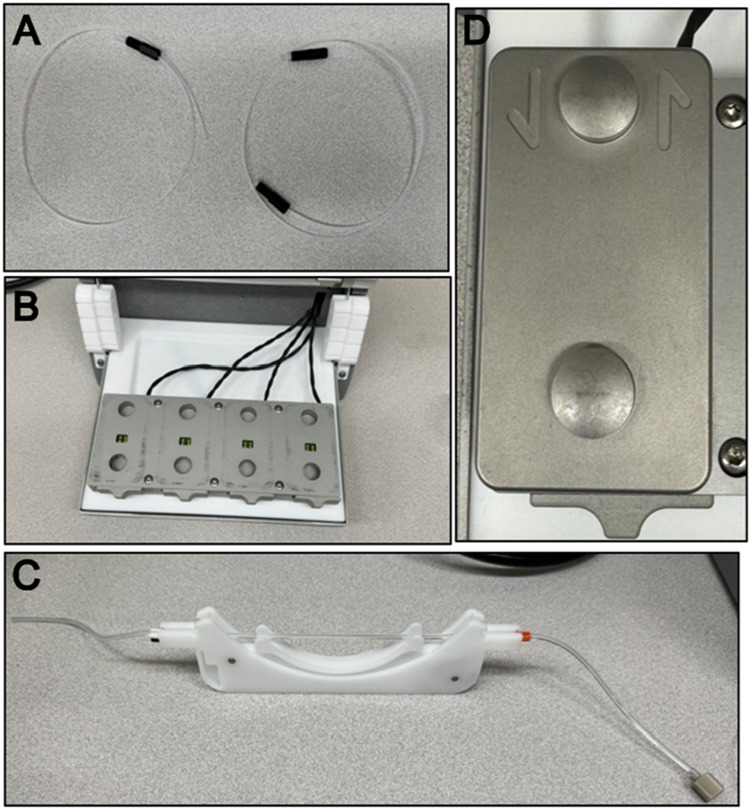
QCM-D apparatus components. **(A)** Left is assembled flow module inlet tubing and right is assembled flow module outlet tubing. **(B)** An empty QCM-D chamber with no flow modules. **(C)** Assembled peristaltic pump cassette and Tygon tubing. **(D)** Top view of a flow module with the inlet and outlet arrows showing directionality for flow and appropriate tubing.

**FIGURE 4:**
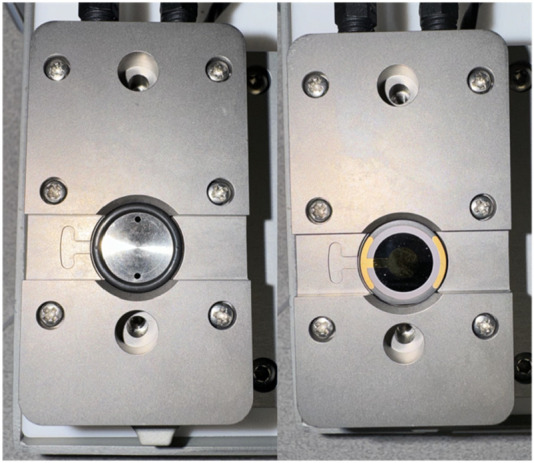
Sensor orientation within the QCM-D module. (Left) An empty QCM-D flow module. (Right) QCM-D flow module with a Biolin Scientific gold sensor properly placed.

**FIGURE 5:**
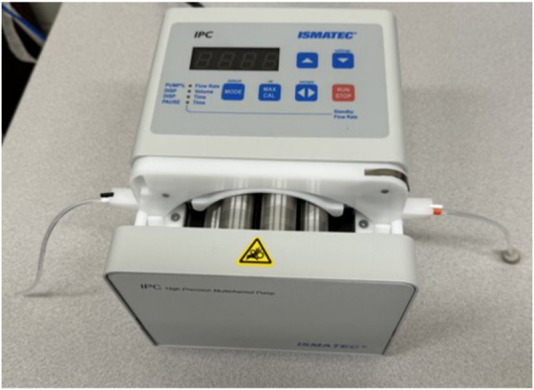
Installation of tubing with pump. Peristaltic pump with the cassette and Tygon tubing installed. Be sure to not crease the tubing.

1. Measure the length from the module’s position in the device to where the sample tubes will sit with the Teflon tubing, cut this tubing at a right angle with the tube cutting device. This will be the inlet tubing.
2. Measure the length from the module’s position in the device to the pump with the Teflon tubing, cut this tubing at a right angle with the tube cutting device. This will be the outlet tubing.
3. Place one nut on the inlet tubing and then a ferrule right after, screw the tubing into one side of the module to allow the ferrule to mold to the tube’s shape. It does not matter which side is chosen. Place one nut on the outlet tubing and then a ferrule right after, screw the tubing into one side of the module to allow the ferrule to mold to the tube’s shape, repeat for the other side of the tubing. An assembled tubing can be seen in Figure 3A.
4. Take one Tygon tube and add a perifit to one side. It does not matter which side is chosen. This is the pump tubing.
5. The module has dedicated inlet and outlet ports, put the inlet and outlet tubes in their dedicated spots, then place the module in the QCM device. The QCM device can be seen in Figure 3B and the module can be seen in Figure 3D.
6. Place the pump tubing inside one of the cassettes. The operator will need to stretch the tubing; this will not damage it. Place the colored markers on each end into the slots of the cassette to hold the tubing in place.
7. Open the module and place it in its position in the QCM-D, carefully place the gold sensor into the module as indicated in Figure 4. Then replace the module top.
  a. To place the sensor only touch the clear/white quartz portion of the sensor and position the sensor in line with the indicator on the module. Refer to Figure 2 to see the proper portion of the sensor to touch and Figure 4 to see proper sensor placement in the module.
8. Place the cassette in the pump as seen in Figure 3C.
9. Place the module into the first position with the electrodes down, slide the locking mechanism at the front of position one to the left to lock the module in place. This positioning and assembly can be seen in Figure 5.
10. Carefully put the inlet and outlet tubing into the tube hooks in the device.
11. Attach the pump tubing and outlet tubing by screwing the nut into the perifit.
  a. Do not pull the inlet or outlet tubing too hard nor move it at any sharp angle at any point. Any creases in the tubing can cause major disruption to the experiment.
12. Put the exit end of the pump tubing in a waste beaker.
  The operator is now ready to begin cleaning the QCM-D.

#### Cleaning the QCM-D

1. Make sure the sensor in the QCM-D is a cleaning sensor following step 8 above
  a. Do not confuse the action of cleaning sensors for experiments with a “cleaning sensor” for preparing the QCM-D. To clean the QCM-D module, any sensor can be used, such as an older or extensively used sensor. The sensor to be used in your experiment, or experimental sensor, must be cleaned with base piranha solution, as indicated above, and should not be scratched, damaged, or extensively used.
2. Turn on the QCM-D and the peristaltic pump
3. Set the pump to 0.300 mL/min flow rate with the up and down arrows
4. Verify the flow is going in the correct direction towards the waste beaker with the escape button
5. Open the QSoft401 software from Biolin Scientific.
6. Click the Temp Ctrl button and set the temperature to 30°C for cleaning
7. Once the Tact, or actual temperature, reaches 30°C, pump approximately 5 mL of 2% Hellmanex II through the device via the inlet tube placed in a beaker
8. Every few minutes, press the max/cal button in order to send the solution through the machine at the maximum flow rate for a few moments.
9. Once all Hellmanex is gone, stop the pump and place the inlet tubing in 5 mL of DI H_2_O, repeat step 7
10. Once all DI H_2_O is gone, remove the tubing from the beaker and pump air through the device until no liquid is visible in the tubes or until no more liquid will come out.
11. Stop the pump once all liquid is gone
12. Disconnect the pump tubing and outlet tubing
13. Adjust the N_2_ tank to 25 psig
14. Remove the cleaning sensor with the tweezers, hold the sensor over a paper towel, and dry the sensor with N_2_ by blowing the liquid down towards the tweezers to avoid contamination
15. Blow the inlet, outlet, and pump tubing out with N_2_ until no liquid is visibly present. Also blow the inside of the module out where the sensor was placed during cleaning for the solution to flow over the sensor.

### QCM-D experiment setup

With cytoskeletal reagents prepared and sensors cleaned, the operator is now ready to set up the QCM-D experiment. This section walks through the physical set-up of the flow module, tubing, flow conditions, and software conditions. Careful handling of components is critical to avoid disrupting flow or introducing bubbles, which can negatively impact frequency and dissipation readings.

1. Carefully place an experimental sensor in the QCM-D module as indicated below. Then replace the module top. Place the module into the first position with the electrodes down, slide the locking mechanism at the front of position one to the left to lock the module in place.
  a. To place the sensor, only touch the clear/white quartz portion of the sensor and position the sensor in line with the indicator on the module as illustrated in Figure 2.
2. Carefully put the inlet and outlet tubing into the tube hooks in the device. Attach the pump tubing and outlet tubing by screwing the nut into the perifit. Do not pull the inlet or outlet tubing too hard nor move it at any sharp angle at any point. Any creases can cause major disruption to the experiment. Make sure the exit end of the pump tubing in a waste beaker.
3. Adjust the pump to the experimental flow rate with the up and down arrows
4. Verify the flow is going in the correct direction towards the waste beaker with the escape button
5. Adjust the temperature to the experimental temperature chosen by clicking Temp Ctrl and typing in the necessary temperature
6. Click Acquisition on the top of the screen then Setup Measurement
7. Under included sensors make sure only one is clicked and the desired harmonics
  a. Usually first through ninth
8. Click ‘Find all’ under the Find all resonances section
  a. Make sure harmonic one reads approximately 32 for the dampening and approximately 250 for amplitude before proceeding. If it does not read close to these values, adjust the sensor inside the module.

Note: Always stop the pump before moving the inlet tubing from one solution to another to avoid the data being contaminated by bubbles and to never click the max/cal button during an experiment.

Note: If the tweezers are still too sharp or tight and damage the sensor, pad the tips with parafilm or the tips of a pair of gloves to further reduce the risk of breakage.

### Actomyosin bundle assembly on the QCM-D sensor

This section details the sequential addition of key proteins and blocking agents onto the QCM-D sensor to assemble an actomyosin bundle. Each component plays a specific role in forming a stable, layered structure that mimics cytoskeletal organization. The experiment begins by establishing a baseline in buffer, followed by deposition of poly-L-lysine (PLL) to promote actin filament binding via electrostatic interactions. Next, actin filaments are introduced to form the core structural layer. A brief incubation with casein helps block nonspecific binding sites before the addition of myosin, which interacts with the actin layer to simulate force-generating interactions. A second addition of actin can enhance filament bundling, and the final buffer wash helps stabilize the complex for data interpretation. Throughout this process, it is critical to document the timing of each step using the note and timestamp features in the QCM-D software. Precise labeling of solution changes allows the operator to correlate shifts in frequency and dissipation with specific molecular events, which is essential for accurate data analysis and interpretation.

Follow the steps below to build the actomyosin assembly directly on the sensor surface under controlled flow and temperature conditions.

1. Set the peristaltic pump flow rate to 0.100 mL/min
2. Set the temperature control on the computer to 25°C
3. Click Acquisition then Start Measurement and the measurement box will appear
4. Place the inlet tubing into the GAB buffer to establish a baseline. This usually takes about 10 min
5. Once the frequency and dissipation readings settle into a horizontal line, click acquisition then restart data collection
6. Click on the “N” and “S1” tabs in order to open up the notes and the graph windows
7. Add the note “0:00 Buffer” in the note window in order to indicate the experiment beginning with a buffer baseline
8. Allow the baseline buffer to run for another 10–15 min to ensure a stable baseline is achieved
9. Pause the pump by clicking the stop button and switch the inlet tube to PLL in GAB. When a change occurs on the graph, click the clock icon in the left corner of the data acquisition window in order to place a time stamp and label it PLL. Allow this measurement to continue until stable measurement occurs or the sample is almost gone*
10. Pause the pump by clicking the stop button and switch the inlet tube to Actin in GAB. When a change occurs on the graph, click the clock icon in the left corner of the data acquisition window in order to place a time stamp and label it Actin. Allow this measurement to continue until stable measurement occurs or the sample is almost gone*
11. Pause the pump by clicking the stop button and switch the inlet tube to Casein in GAB. When a change occurs on the graph, click the clock icon in the left corner of the data acquisition window in order to place a time stamp and label it Casein. Allow this measurement to continue until stable measurement occurs or the sample is almost gone*
12. Pause the pump by clicking the stop button and switch the inlet tube to Myosin in GAB. When a change occurs on the graph, click the clock icon in the left corner of the data acquisition window in order to place a time stamp and label it Myosin. Allow this measurement to continue until stable measurement occurs or the sample is almost gone*
13. Pause the pump by clicking the stop button and switch the inlet tube to Actin in GAB. When a change occurs on the graph, click the clock icon in the left corner of the data acquisition window in order to place a time stamp and label it Actin. Allow this measurement to continue until stable measurement occurs or the sample is almost gone*
14. Pause the pump by clicking the stop button and switch the inlet tube to GAB. When a change occurs on the graph, click the clock icon in the left corner of the data acquisition window in order to place a time stamp and label it Buffer. Allow this measurement to continue until stable measurement occurs or the sample is almost gone*
15. Click Acquisition then Stop Data Collection
16. Proceed with the QCM-D cleaning protocol and Data Analysis

*Note: It usually takes 2–4 min between moving the inlet tube to a new sample and observing changes on the graph at this flowrate.

### Actomyosin bundle assembly with ADP wash

This section builds upon the standard actomyosin assembly protocol by introducing an ADP wash at the end of the experiment to investigate how the nucleotide state of myosin alters the mechanical properties of the assembled cytoskeletal bundle. Myosin’s interaction with actin is nucleotide-dependent: in the ATP-bound state, myosin has a low affinity for actin and detaches easily, whereas in the ADP-bound state, myosin binds actin more readily. Introducing ADP after bundle formation allows us to examine how these biochemical changes influence actomyosin network mechanics in real time, as observed through QCM-D frequency and dissipation shifts. Throughout this process, it is essential to use the notes and timestamp features in the QCM-D acquisition software to precisely document when each solution is introduced. This ensures that observed changes in the data can be accurately matched to specific experimental steps, which is especially important when analyzing subtle shifts in mechanical properties due to nucleotide state transitions.

1. Set the peristaltic pump flow rate to 0.100 mL/min
2. Set the temperature control on the computer to 25°C
3. Click Acquisition then Start Measurement and the measurement box will appear
4. Place the inlet tubing into the GAB buffer to establish a baseline. This usually takes about 10 min
5. Once the frequency and dissipation readings settle into a horizontal line, click acquisition then restart data collection
6. Click on the “N” and “S1” tabs in order to open up the notes and the graph windows
7. Add the note “0:00 Buffer” in the note window in order to indicate the experiment beginning with a buffer baseline
8. Allow the baseline buffer to run for another 10–15 min to ensure a stable baseline is achieved
9. Pause the pump by clicking the stop button and switch the inlet tube to PLL in GAB. When a change occurs on the graph, click the clock icon in the left corner of the data acquisition window in order to place a time stamp and label it PLL. Allow this measurement to continue until stable measurement occurs or the sample is almost gone*
10. Pause the pump by clicking the stop button and switch the inlet tube to Actin in GAB. When a change occurs on the graph, click the clock icon in the left corner of the data acquisition window in order to place a time stamp and label it Actin. Allow this measurement to continue until stable measurement occurs or the sample is almost gone*
11. Pause the pump by clicking the stop button and switch the inlet tube to Casein in GAB. When a change occurs on the graph, click the clock icon in the left corner of the data acquisition window in order to place a time stamp and label it Casein. Allow this measurement to continue until stable measurement occurs or the sample is almost gone*
12. Pause the pump by clicking the stop button and switch the inlet tube to Myosin in GAB. When a change occurs on the graph, click the clock icon in the left corner of the data acquisition window in order to place a time stamp and label it Myosin. Allow this measurement to continue until stable measurement occurs or the sample is almost gone*
13. Pause the pump by clicking the stop button and switch the inlet tube to Actin in GAB. When a change occurs on the graph, click the clock icon in the left corner of the data acquisition window in order to place a time stamp and label it Actin. Allow this measurement to continue until stable measurement occurs or the sample is almost gone*
14. Pause the pump by clicking the stop button and switch the inlet tube to GAB. When a change occurs on the graph, click the clock icon in the left corner of the data acquisition window in order to place a time stamp and label it Buffer. Allow this measurement to continue until stable measurement occurs or the sample is almost gone*
15. Pause the pump by clicking the stop button and switch the inlet tube to ADP Wash. When a change occurs on the graph, click the clock icon in the left corner of the data acquisition window in order to place a time stamp and label it ADP Wash. Allow this measurement to continue until stable measurement occurs or the sample is almost gone*
16. Click Acquisition then Stop Data Collection
17. Proceed with the QCM-D cleaning protocol and Data Analysis

*Note: It usually takes 2–4 min between moving the inlet tube to a new sample and the change occurring on the graph.

### QCM-D data analysis

Once data collection is complete, the next step is to analyze the QCM-D output to measure how each biomolecular layer influences the mechanical properties of the system. The QCM-D measures changes in resonance frequency (Δf) and energy dissipation (ΔD) of the sensor crystal, which correlate with mass adsorption, viscoelastic properties, and layer thickness. By applying appropriate models in the QSense Dfind software, one can extract physical parameters such as elastic modulus, viscosity, and film thickness, providing insight into how different components of the system contribute to the overall mechanics. However, extraction of these parameters uses a model based off the Sauerbrey equation that assumes a uniform, homogeneous film (Kerivan et al., 2024; Kerivan et al., 2023). In this protocol, the actomyosin “coating” is heterogeneous in both composition and surface coverage, which violates these assumptions and prevents accurate modeling of separate viscosity and elasticity contributions. As a result, until more robust models are developed that can account for such heterogeneous and soft films, interpretation is limited to qualitative trends in the raw frequency and dissipation shifts, without assigning quantitative changes to viscosity or elasticity alone. Further data analysis can involve selecting appropriate harmonics with minimal noise, applying model fitting tools such as Smartfit or Broadfit, and assessing model quality to ensure accurate interpretation. Depending on the experimental conditions and data quality, the operator may need to adjust the baseline, exclude noisy harmonics, or compare outputs across different modeling approaches. Throughout the process, the operator can generate visual outputs of raw and fitted data, and export figures for presentation or publication.

**FIGURE 6:**
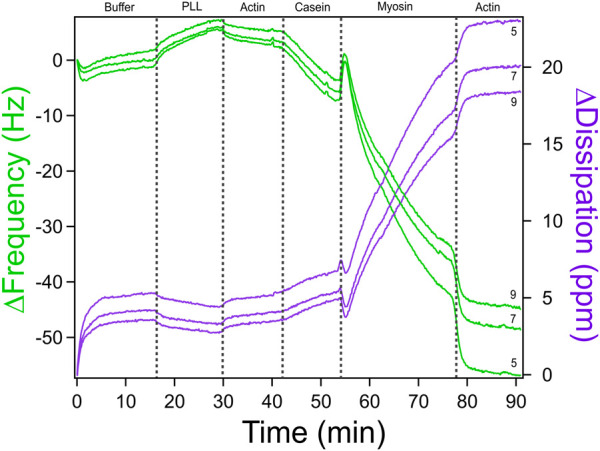
QCM-D Measurements of Actomyosin Bundle Assembly with Multiple Overtones. A representative QCM-D measurement of change in frequency (green) and change in dissipation (purple) versus time for assembly of an actomyosin bundle. Measurements at overtones 5, 7, and nine are shown for comparison. Overtones less than five and greater than nine are typically noisy. For subsequent analysis, it is recommended to choose the harmonic overtone with the optimal signal-to-noise ratio. We chose overtone seven for our analysis. Adapted with permission from Kerivan et al. (2024) Cellular and Molecular Bioengineering under license number 6071410087688.

1. Open the QSense Dfind software and select ‘Create a new project’. Select the. qsd file from the experiment.
2. A dialog box will automatically open prompting the operator to establish period borders for the data. Select ‘notes taken during data acquisition’ and then exclude any extra labels that will not be relevant for the result graphs.
3. A df(t) vs. dD(t) graph will be generated. On the bottom left deselect harmonics 1, 3, 9, and 11 as they usually have a lot of interference. Harmonics five and seven typically have significantly less noise, as seen in Figure 6. Choose the one with the least amount of interference.
4. Drag the baseline to include the entirety of the buffer baseline run
5. Select the Model/Layers tab at the top of the screen. Select these models needed for analysis:
  a. Smartfit
  b. Modeled f&D of S1
6. On the Smartfit model look to the top right to be sure that the fit quality is good. Hover over each dot to determine this. If it is >0.9, then the fit is good
  a. A green dot is a high model fit, yellow is a medium model fit, and red is a low model fit
7. On the Smartfit model the operator will see four graphs: thickness, chi square, elastic modulus, and viscosity. Use these to gather data about layers being added on the surface of the sensor.
8. If the fit quality is <0.9, it is a bad fit. Go back to the Model/Layers tab and select Broadfit to attempt for a better fit.
  a. A green dot is a high model fit, yellow is a medium model fit, and red is a low model fit
9. On the Broadfit model, the operator will see four graphs: thickness, chi square, elastic modulus, and viscosity. Use these to gather data about layers being added on the surface of the sensor.
10. After data has been gathered from either Smartfit or Broadfit, return to the Model/Layers tab and select ‘Comparing Layer Models’ in order to obtain a graph with a full comparison of the models versus the original raw data
11. For any graphs the operator would like to save, right click, select ‘export’, and then ‘Figure with transparent background’

## Anticipated results

### Actomyosin bundle assembly and mechanical behavior

**FIGURE 7:**
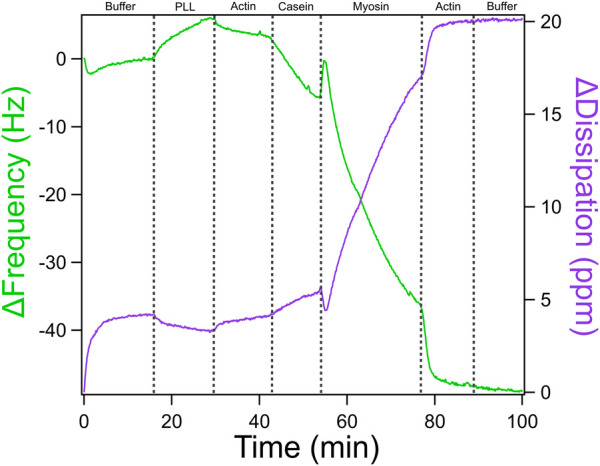
QCM-D Trace of Actomyosin Assembly with ATP-based Buffer. QCM-D measurements showing additions of an ATP-based buffer solution, PLL, actin, casein, and myosin in order to assemble an actomyosin bundle on the gold-coated piezoelectric sensor. Adapted with permission from Kerivan et al. (2024) Cellular and Molecular Bioengineering under license number 6071410087688.

A quartz crystal microbalance with dissipation monitoring (QCM-D) was used to evaluate the formation of actomyosin bundles to understand the molecular factors that influence emergent mechanics within the actomyosin cytoskeleton. We performed a stepwise assembly of the actomyosin bundle on the gold sensor surface with real time monitoring of changes in frequency (Δf) and dissipation (ΔD). The QCM-D experiment was conducted at 25°C and 0.100 mL/min with careful switching of solutions in order to avoid the introduction of air bubbles which would have caused interference and noise to the data. A representative QCM-D trace is shown in Figure 7 with distinct shifts in frequency and dissipation corresponding to each addition of PLL, actin, casein, myosin, actin, a final GAB buffer addition. QCM-D data provides real-time information about how molecular layers assemble and evolve on a surface. The two main outputs, frequency (Δf) and dissipation (ΔD), offer insight into both the mass of material binding to the sensor and the mechanical properties of the resulting layers. A decrease in frequency reflects mass deposition on the sensor surface, while an increase in dissipation indicates that the newly formed layer is compliant. In contrast, a decrease in dissipation suggests that the layer has become more rigid. During actomyosin bundle assembly, these signals can be used to interpret how each component addition influences system mechanics at each stage of the experiment.

Moving from left to right across the QCM-D trace, each component added to the sensor surface produces a characteristic shift in frequency (Δf) and dissipation (ΔD), which together provide a mechanical and structural “signature” of that step. The experiment begins with the introduction of poly-L-lysine (PLL), which shows only a modest change in frequency and dissipation. This indicates that a thin layer of mass is adsorbed onto the surface and that it remains relatively rigid and compact. A slight increase in frequency was sometimes observed upon buffer and PLL additions. These shifts likely result from the removal of loosely bound solutes or minor changes in hydrodynamic coupling to the sensor surface. The first addition of actin results in a slightly larger drop in frequency and a modest increase in dissipation, confirming that filaments are binding and that the surface is beginning to soften due to the flexible nature of the actin network. The casein layer produces a more substantial shift. Frequency drops sharply, and dissipation increases significantly. This reflects the addition of more disordered, hydrated protein mass and suggests that casein is filling in open spaces, making the layer more viscoelastic and loosely packed. When myosin is introduced, the frequency again decreases sharply, consistent with mass binding to the existing actin network. At the same time, dissipation rises, revealing that myosin binding promotes structural rearrangement and further softens the surface. A second addition of actin leads to another decrease in frequency, indicating more filament binding, but only a modest increase in dissipation. This suggests that the system is nearing structural saturation where new actin filaments integrate but do not substantially alter the overall softness of the bundle. In Figure 7, a high concentration of myosin promotes more bundle formation. If the myosin concentration were lowered, we would expect reduced crosslinking, resulting in softer, less mechanically stable bundles. This progression of data highlights how QCM-D can sensitively resolve both binding events and changes in mechanical properties at each stage of actomyosin bundle formation, providing a powerful tool to dissect the structural contributions of individual cytoskeletal components.

### Effect of an ADP wash on the actomyosin bundle

**FIGURE 8:**
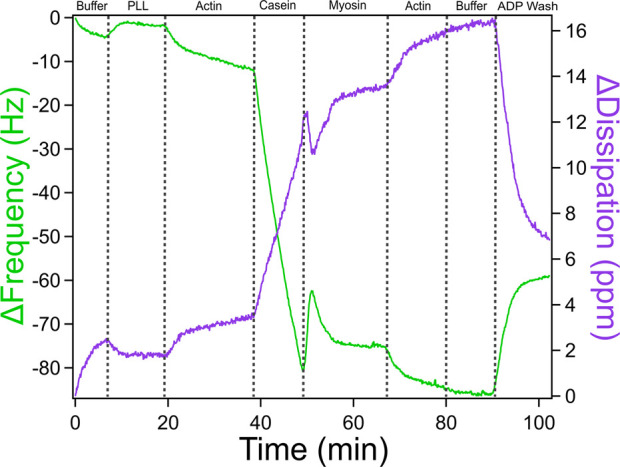
QCM-D Trace of Actomyosin Bundle Assembly with an ADP-based Buffer Wash. QCM-D measurements showing the assembly of an actomyosin bundle on top of a piezoelectric sensor with an additional ADP wash at the end to measure the mechanical changes caused by altering motor activity. Adapted with permission from Kerivan et al. (2024) Cellular and Molecular Bioengineering under license number 6071410087688.

To better understand how ATP hydrolysis influences the stability and mechanics of actomyosin bundles, an ADP wash was introduced following bundle assembly (see Figure 8). Myosin operates through an ATPase cycle in which its binding affinity for actin varies depending on the bound nucleotide. In the presence of ATP, myosin detaches from actin and undergoes conformational changes throughout the hydrolysis cycle, influencing motor activity and filament sliding. In contrast, in the ADP-bound state, myosin binds more tightly to actin. This shift not only alters myosin’s grip on actin but changes actin network architecture and stiffness, which in turn feeds back to regulate myosin behavior. Introducing ADP into the system after bundle formation allows probing of this interdependence by observing how the nucleotide state affects actomyosin connectivity, mass retention, and viscoelasticity as captured by QCM-D frequency and dissipation shifts. The ADP wash in Figure 8 resulted in a steep frequency increase and dissipation decrease indicating a significant increase in the rigidity of the actomyosin bundle. Unlike the higher myosin concentration in Figure 7, which likely favored denser crosslinking, the conditions in Figure 8 contain a lower myosin concentration by half, which may contribute to more pronounced mechanical changes observed following the addition of an ADP wash. While we cannot fully exclude the contribution of ADP accumulation from prior ATP hydrolysis, the experimental design, specifically, the timed and controlled addition of ADP following a defined incubation period with ATP, allows us to isolate the effect of ADP introduction. The distinct shift in QCM-D signals observed after the ADP wash, compared to the previous ATP-driven incubation, supports the interpretation that the observed stiffening primarily reflects the effect of externally introduced ADP rather than residual hydrolysis products. For more in depth discussion of data analysis, mechanistic interpretation, and additional traces, see Kerivan and Amari et al. (Kerivan et al., 2024).

To interpret these results, it is important to consider the myosin ATPase cycle. Myosin’s interaction with actin depends on its nucleotide state. In the presence of ATP, myosin detaches from actin, enabling filament sliding and network remodeling. This dynamic activity likely explains the observed increase in dissipation upon ATP addition, reflecting a more compliant, actively restructured actomyosin network. In contrast, in the ADP-bound state, myosin binds tightly to actin, leading to reduced motor activity and a more rigid, static bundle, evidenced by decreased dissipation. The concurrent increase in frequency suggests a net reduction in effective mass on the sensor, potentially due to the stiffened bundle contracting and partially disengaging from the surface. Together, these findings support a model in which ATP-driven myosin activity maintains mechanical flexibility and surface contact, while ADP shifts the system toward a rigid, less dynamic state. This underscores the reciprocal interplay between nucleotide state, actomyosin mechanics, and bundle architecture.

## Discussion and outlook

QCM-D offers a powerful and underutilized platform for probing the mechanical dynamics of reconstituted cytoskeletal systems. Its ability to simultaneously monitor mass adsorption and viscoelastic properties in real time makes it uniquely suited for studying emergent behaviors in actomyosin networks that are otherwise difficult to capture through traditional techniques (Gagliardi et al., 2023; Tonda-Turo et al., 2018; Kushiro et al., 2016). In this protocol, we employed gold-coated QCM-D sensors treated with poly-L-lysine (PLL) in General Actin Buffer (GAB) to enable electrostatic adsorption of actin filaments. While PLL does not form covalent bonds with gold, this simple and rapid surface preparation method provided sufficient actin attachment to support the formation and mechanical remodeling of actomyosin bundles. However, the use of PLL on gold surfaces may contribute to variability in surface coverage and bundle formation due to non-specific interactions and less stable protein adhesion compared to self-assembled monolayers (SAMs) or PEGylated coatings (Biolin, 2021; Bianco et al., 2018). One trade-off of using QCM-D is the relatively high amount of protein required for each experiment due to the multi-milliliter-sized volumes necessary for the microfluidic setup. Additionally, the full experimental workflow, from sensor preparation and coating to flow equilibration and data collection, can take several hours per condition, limiting throughput for large screening applications. Despite these limitations, QCM-D measurements are reproducible when experimental parameters such as buffer composition, temperature, and flow rate are carefully controlled. Minor variations in dissipation (ΔD) and frequency (Δf) responses across replicates likely reflect subtle differences in actin filament length, surface coverage, or myosin motor activity. These observations highlight the importance of optimizing flow conditions, incubation times, and coating protocols to ensure consistent bundle formation and network remodeling (Bianco et al., 2018; Dumont and Prakash, 2014; Finer et al., 1994).

Importantly, QCM-D measurements can complement existing biophysical techniques by providing a mesoscopic view of cytoskeletal mechanics. Optical trapping enables precise quantification of force generation by individual myosin motors or small ensembles, while fluorescence microscopy offers high-resolution visualization of actin filament dynamics and myosin-driven contraction (Al Azzam et al., 2021; Biolin Scientific, 2024; Kwon and Dodge, 2015; Al Azzam O. Y. et al., 2022; Kaya et al., 2017; Liu et al., 2018). Atomic force microscopy (AFM) adds yet another layer of insight by directly measuring nanoscale stiffness and topography of cytoskeletal structures (Kerivan et al., 2024; Tagaya, 2015). QCM-D bridges these approaches by detecting system-wide mechanical transitions, such as viscoelastic stiffening, relaxation, or collapse, in real time and without the need for labeling (Gagliardi et al., 2023; Kushiro et al., 2016). QCM-D could also be integrated with advanced imaging techniques using a window module, enabling simultaneous fluorescence microscopy during mechanical measurements, allowing researchers to correlate structural dynamics with mechanical changes in real time (Kerivan et al., 2023).

The ability of QCM-D to sensitively track changes in network mechanics also holds promise for disease-related studies. Aberrant actomyosin interactions underlie a range of pathological processes, including altered contractility in metastatic cancer cells and impaired motor activity in neurodegenerative diseases such as Alzheimer’s and Parkinson’s (Kerivan et al., 2023; Al Azzam et al., 2021; Kushiro et al., 2016; Luo et al., 2013; Wagoner and Dill, 2021). QCM-D has already been used to detect softening or stiffening of reconstituted or cell-derived cytoskeletal structures in response to genetic mutations or pharmacological perturbations, and similar approaches could be expanded to include disease-relevant motor protein isoforms or cytoskeletal regulators (Kushiro et al., 2016; Ruegg et al., 2002).

As applied in this protocol, QCM-D can monitor real-time mechanical remodeling in reconstituted systems, making it well-suited for studying the mechanical properties and feedback behavior of proteins during cytoskeletal assembly, motor-driven contractility, and disease-related perturbations. Although QCM-D is capable of extracting viscoelastic parameters, accurate quantification relies on assumptions of a uniform and homogeneous film (Kerivan et al., 2024; Kerivan et al., 2023). In the present protocol, the actomyosin coating is heterogeneous in both composition and surface coverage, violating these assumptions and preventing reliable modeling of distinct viscosity and elasticity components. As a result, we interpret the raw frequency and dissipation shifts qualitatively, without assigning specific changes to either viscoelastic parameter. Future work aimed at developing models that account for soft, heterogeneous films would greatly enhance the ability to derive quantitative mechanical insights from such systems. Nevertheless, when integrated with complementary techniques such as force spectroscopy and advanced imaging, QCM-D contributes to a multimodal framework for probing the biophysical foundations of cellular structure and function. Its experimental flexibility, through modifiable surface coatings, protein assemblies, and buffer conditions, enables precise control and broad applicability. This adaptability supports extensions beyond actomyosin networks to include microtubules, motor proteins, and crosslinkers, as well as variable environmental factors such as ionic strength and macromolecular crowding, facilitating comprehensive exploration of cytoskeletal mechanics.

## Conclusion

This study presents a detailed protocol for utilizing Quartz Crystal Microbalance with Dissipation (QCM-D) in conjunction with an actomyosin assay to investigate the dynamic interactions between actin filaments and myosin motor proteins. By leveraging QCM-D’s high sensitivity to mass and viscoelastic changes at the nanoscale, we provide a robust approach to characterizing actomyosin binding kinetics, contractile forces, and filament organization in real time (Kerivan et al., 2024; Gagliardi et al., 2023; Kushiro et al., 2016). The QCM-D integration with the actomyosin assay offers key advantages, including increased understanding of molecular interactions and the ability to distinguish between mass accumulation and structural reorganization (Biolin, 2021; Tagaya, 2015; Tonda-Turo et al., 2018). These findings complement existing approaches such as optical trapping and fluorescence microscopy, expanding the biophysical toolkit for understanding cytoskeletal mechanics and motor protein function (Al Azzam et al., 2021; Kwon and Dodge, 2015;Kaya et al., 2017).

## Data Availability

The original contributions presented in the study are included in the article/supplementary material, further inquiries can be directed to the corresponding author.
